# Unexpected complexity of the Reef-Building Coral *Acropora millepora *transcription factor network

**DOI:** 10.1186/1752-0509-5-58

**Published:** 2011-04-28

**Authors:** Taewoo Ryu, Charalampos Harris Mavromatis, Till Bayer, Christian R Voolstra, Timothy Ravasi

**Affiliations:** 1 Division of Chemical & Life Sciences and Engineering and Division of Applied Mathematics and Computer Science, King Abdullah University of Science and Technology, Thuwal, Kingdom of Saudi Arabia

## Abstract

**Background:**

Coral reefs are disturbed on a global scale by environmental changes including rising sea surface temperatures and ocean acidification. Little is known about how corals respond or adapt to these environmental changes especially at the molecular level. This is mostly because of the paucity of genome-wide studies on corals and the application of systems approaches that incorporate the latter. Like in any other organism, the response of corals to stress is tightly controlled by the coordinated interplay of many transcription factors.

**Results:**

Here, we develop and apply a new system-wide approach in order to infer combinatorial transcription factor networks of the reef-building coral *Acropora millepora*. By integrating sequencing-derived transcriptome measurements, a network of physically interacting transcription factors, and phylogenetic network footprinting we were able to infer such a network. Analysis of the network across a phylogenetically broad sample of five species, including human, reveals that despite the apparent simplicity of corals, their transcription factors repertoire and interaction networks seem to be largely conserved. In addition, we were able to identify interactions among transcription factors that appear to be species-specific lending strength to the novel concept of "Taxonomically Restricted Interactions".

**Conclusions:**

This study provides the first look at transcription factor networks in corals. We identified a transcription factor repertoire encoded by the coral genome and found consistencies of the domain architectures of transcription factors and conserved regulatory subnetworks across eumetazoan species, providing insight into how regulatory networks have evolved.

## Background

Deciphering and predicting transcriptional regulatory networks is of considerable importance in understanding how organisms function, adapt, and respond to changes in their environment. Much effort has been addressed to elucidate these regulatory networks in several model organisms. For instance, global transcription factors (TFs) combinatorial interaction maps were built in human and mouse [1] and developmental gene regulatory circuits were elucidated in the sea urchin embryo [2,3]. However, little effort has been made so far in understanding the structure, function, and conversation of transcriptional networks in non-model organisms, e.g. corals, despite their ecological importance.

Corals are members of the phylum Cnidaria that includes such diverse forms as jellyfish, hydra, and sea anemones. Reef-building corals (Cnidaria: Hexacorallia: Scleractinia) in symbiosis with their unicellular photosynthetic dinoflagellate symbionts (Alveolata: Dinophycea: *Symbiodinium*) provide the foundation of coral reef ecosystems, and are well known for providing biodiversity to marine ecosystems [4]. The sensitivity of corals to environmental stresses such as temperature, salinity, and nutrient loading make them highly vulnerable to climate change, ocean acidification, and other anthropogenic impacts [5]. As a consequence, coral cover has continuously declined in recent decades [6]. A more detailed understanding of how scleractinian corals and their associated microbes will respond to environmental changes is needed in order to eventually establish effective management policies that are able to conserve and sustain coral reef ecosystems. So far only a few studies have looked at the mechanisms on a molecular level [7-12] that go beyond transcriptome annotation and ortholog identification [13,14]. The emerging picture from these studies is that corals are complex organisms as revealed by a diverse set of receptors and a comprehensive innate immunity reservoir which are important for responses to the environment [15].

In this study, we developed and applied a systems-wide integrative approach to assess the complexity of the *Acropora millepora *transcription factor (TF) network by reconstructing a TF interaction map from known interactions and comparing it to those of four model organisms (fruitfly, sea urchin, mouse, and human). The *A. millepora *TF repertoire was identified using sequence-specific DNA binding domains from DBD [16]. Subsequently conserved combinatorial TF interactions as well as species-specific TF interactions were inferred by the integration of the fly, mouse and human TFs interaction networks [1,17] followed by phylogenetic network footprinting analysis. Our study provides not only the first comprehensive catalog of *A. millepora *TFs but also a first assessment of how these are organized to form transcriptional networks. Evidence of conservation and divergence across the phylogenetic tree can also be inferred from this analysis. The analysis presented here can be considered a starting point for a more comprehensive study of regulatory networks in corals based on coral reef genomics- and systems biology-based interpretive framework.

## Results and Discussion

### The *A. millepora *TF repertoire

**Figure 1:**
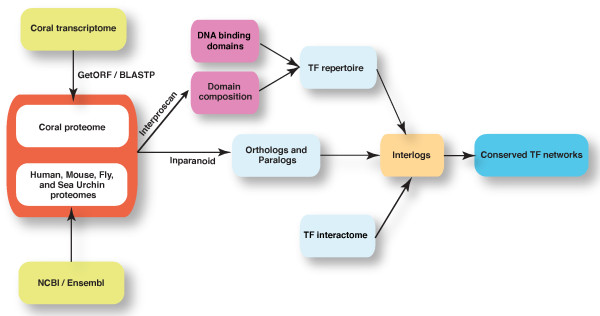
**Workflow showing our systematic approach used to infer species-specific transcription factor networks across eumetazoan animals**.

Recently, the planulae transcriptome of the staghorn coral *A. millepora *was published using next generation sequencing technology [13]. Even though comprehensive information such as gene composition, gene ontology, and associated signaling/metabolic pathways are revealed, detailed mechanisms for the understanding of coral-specific function including the TFs repertoire and their interaction to form transcriptional networks remain to be elucidated. In this study, the TF repertoire and interaction network in *A. millepora *were computationally surveyed using a unique experiment-derived TFs interactome [1] and a phylogenetic network footprinting approach [18] as shown in Figure 1.

Firstly, all possible open reading frames (ORF) for each contig and singleton were identified and translated by applying GetORF [19]. To identify coral proteins whose orthologs exist in other species, all coding sequences were queried against NCBI non-redundant (nr) database using BLASTp [20], the best-hit sequence for each contig or singleton was chosen. If no best hit was found, the longest coding sequence of contig/singleton was chosen. The latter cases are most likely coral-specific proteins or new proteins currently uncharacterized. 19,840 and 49,320 protein sequences were chosen from 104,005 contigs/singletons in coral transcriptome data by abovementioned two approaches, respectively.

To identify the transcription factor repertoire we used 147 sequence-specific DNA binding domains which have been manually curated recently [16]. Protein sequences of the five analyzed species (*Acropora millepora*, *Drosophila melanogaster*, *Strongylocentrotus purpuratus*, *Mus musculus*, and *Homo sapiens*) were searched by InterProScan to identify those with DNA binding domain signatures [21]. Any protein was regarded as a TF if it had at least one such domain. This approach yielded 359 TF signatures in coral, 1,047 in fruitfly, 839 in sea urchin, 1,462 in mouse, and 1,885 in human (see Additional files 1, 2, 3, 4, and 5).

### Evolutionary signatures of the *A. millepora *transcription factors repertoire

In order to define the evolutionary signatures of the *A. millepora *TF repertoire, we analyzed the protein domain composition of the TFs across the five species. We scanned, the full-length protein sequences of all the TFs using the Pfam database [22]. The 359 TFs identified in *A*. *millepora *contained 60 Pfam domains. We also compared the domain composition of *A. millepora *with that of the fruit fly (1,047 TFs with 123 domains), sea urchin (839 TFs with 157 domains), mouse (1,462 TFs with 166 domains) and human (1,885 TFs with 168 domains), (see Additional file 6).

**Figure 2:**
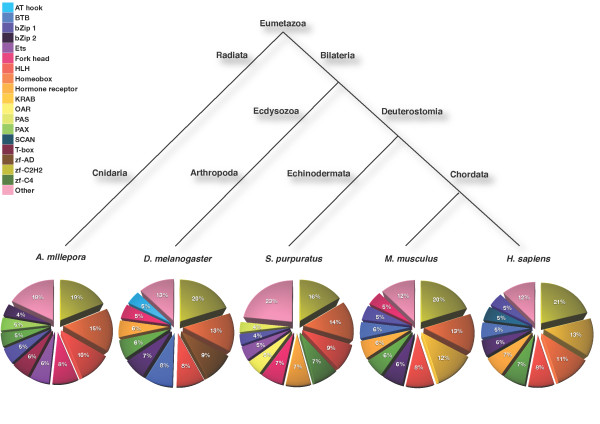
**Phylogenetic relationship between five species and domain architectures**. Pie charts on the bottom show the percentages of the top ten TF domains for each organism. Even though *A. millepora *diverged very early and is morphologically different, it shows a similar domain composition as other lineages and appears as complex as highly evolved bilaterian organisms according to the domain architecture.

Interestingly, the ten most common domains in each species were highly conserved across animal phylogeny (i.e. all five organisms) (Figure 2). However, some domains seemed to be more abundant in coral or marine organisms (i.e. sea urchin and coral) in comparison to the other species (i.e. terrestrial organisms). Ets and Fork head were more frequent in the TF repertoire of coral and sea urchin compared to the fly, mouse, and human. Common life style characteristics might result in such similarities, but further analyses are needed to elucidate the underlying factors that cluster the organisms in these groups. T-box domains were more abundant in coral than other species. Interestingly, proportion of hormone receptor domain is quite low in coral. Four coral TFs contain this domain while fruit fly, sea urchin, mouse, and human have 39, 56, 60, and 101 TFs containing this domain, respectively (see Additional file 6). Hormone receptor domains code for a component of steroid or nuclear hormone receptor TFs (function and origin reviewed in [23]). Even though the origin of steroid receptors has been placed during late stage of deuterostome divergence so far [23], our data suggest that steroid receptors, or at least their functional domains, might have emerged presumably before the divergence of radiata and bilateria (Figure 2). As discussed in a previous study [24], the results of protein family domains analyses have evolutionary implications and can be used to describe evolutionary common life styles and characteristics. Here, we applied an arcsine transformation to the domain distribution in order to reconstruct the evolutionary relationships across the five species [25]. The resulting phylogenetic tree placed each species according to the symmetry of their body design, going from radial to bilateral symmetry, and further on from protostomes to deuterostomes (Figure 2). Hence, our TF domain-based phylogeny recapitulates the established evolutionary relationships between species.

### Inferring *A. millepora *transcriptional network using phylogenetic network footprinting

**Figure 3:**
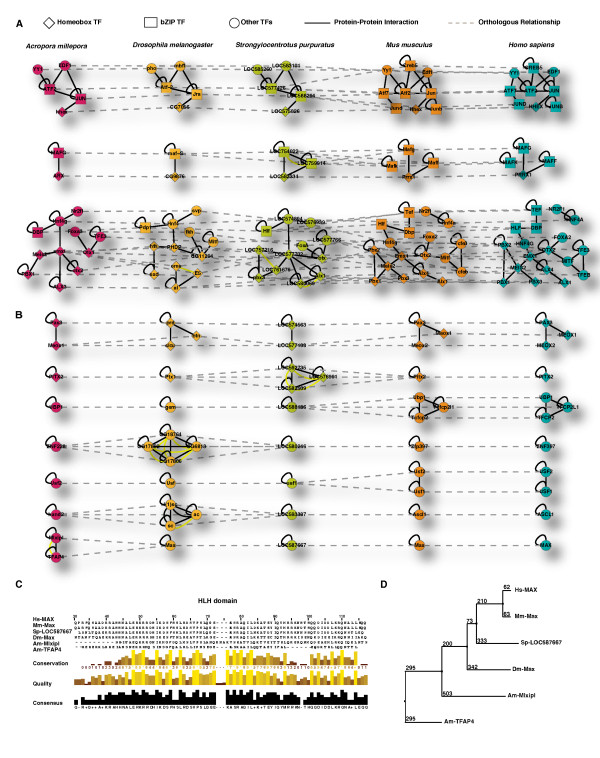
**Conserved transcription factor (sub-) networks in eumetazoa**. Conserved TF interactions across five eumetazoan species were identified and example subnetworks are shown. Presence of these interactions in all five species implies they existed in the last common ancestor before divergence of Radiata and Bilateria. A) The TF network mainly comprised of Homeobox and bZIP TFs. Organisms are ordered according to evolutionary distance. Orthologous relations (dashed lines) are drawn only between adjacent organisms for brevity. Loops indicate homodimeric interaction. B) Notable conserved interactions: Additional homeobox protein interactions essential in early development of all eumetazoans and several TF interactions expanded in particular lineages are drawn. Species order and TF shapes in B are as in A. C) Helix-loop-helix domain in MAX proteins are aligned by Clustal × [44] and conservation in each residue is depicted. D) Phylogeny of MAX proteins estimated by the Mega 3 package [45] using the maximum-parsimony distance. Numbers along branches refer to bootstrapped values.

Little is known about transcriptional networks in marine organisms. This is especially true for corals and to a lesser extent for the sea urchin that has been studied with a view to learn more about embryonic transcriptional networks [2], especially if compared to well-studied organisms such as human and mouse. It is widely accepted that for a given pair of interacting proteins, their corresponding orthologs in other species also interact with each other and this propensity is stronger for highly connected proteins [18,26-28]. These conserved interaction pairs across species, referred to as interlogs, are at the conceptual center of many comparative studies [18,26-28]. Here, for the first step of interlog inference, we identified ortholog groups for each pair of the five species using the Inparanoid algorithm [29] that is known as the most accurate algorithm for true ortholog finding [29,30]. We collected 5,238 human and 1,145 mouse experimentally verified TF interactions from recently published mammalian 2 hybrid assay [1] and 45 fly TF interactions from DIP [17] to construct a TF interaction dataset that represented our source network for the following analysis. All orthologs for a given known interacting TF pair (A-B) were retrieved from Inparanoid result to infer interlogs for the experimentally verified TF dataset source interactions. All possible ortholog combinations of A and B were assumed to interact with each other. This occasionally yielded multiple interlogs from single source interactions, e.g. we retrieved one-to-many or many-to-many ortholog relationships as seen in Figure 3. We consequently interpreted these cases as network expansions. These ortholog relationships are usually the result of gene duplication after speciation events, and this property is specifically captured by the Inparanoid algorithm [31]. We deemed those interactions as true interactions for the following reasons. First, it is widely accepted that one primary source of new genes in eukaryotes is gene duplication followed by divergence. The interacting partners are usually retained between paralogs [32,33]. Second, paralogs duplicated after speciation event (i.e. inparalogs) generally share similar functions and interactions compared to paralogs that were duplicated before speciation events (i.e. outparalogs) [31,34]. Consequently, inparalogs were deemed as true interactions. During above procedure, TFs without any sequence-specific DNA binding domains were excluded. Our approach therefore assures reliable interaction networks by integrating experimentally proven TF interactions, sequence similarity, and functional units of TFs together in a single framework. We were able to infer a total of 3,985 TF interactions with this phylogenetic network footprinting approach. The total number of interactions including source and inferred interactions were 5,509 for human, 2,323 for mouse, 524 for sea urchin, 599 for fruitfly, and 134 for coral (see Additional files 7, 8, 9, 10, and 11). Finally, protein interaction networks were aligned across the five organisms, and conserved TF interactions were identified (see Additional files 12, 13, 14, 15, and 16). The resulting conserved interactions provide the first insight into the structure and properties of combinatorial transcriptional networks in coral and sea urchin (Figure 3A and 3B).

### *A. millepora *transcriptional network shows properties of those of Bilaterian organisms

A close analysis of the conserved transcriptional network depicted in Figure 3A reveals surprising and intriguing properties. *A. millepora *organization of TFs into regulatory networks is similar to those of bilateral organisms. For example *A. millepora *has a homeobox gene regulatory sub-network, comprised of several Hox genes, that is conserved across evolution. In general, syntenic occurrence of these Hox genes was considered a prerequisite for the correct function of these transcription factors in animals [35]. However, disintegration of Hox clusters has been observed in diverse taxa including several cnidarians, and thus the evolutionary significance of synteny regions is in question [36-40]. We wonder if interaction retention between Hox genes might be more important than merely close physical linkage of them. More specifically, one of the larger and most conserved transcriptional networks in *A. millepora *was identified among Hox gene regulatory sub-networks (Figure 3A), and this network is enriched for genes that are implicated in developmental process such as neural tube formation in mammals. Furthermore, there is evidence that this network expanded during mammalian divergence. This probably represents a net gain in modularity and plasticity for the network in more complex organisms (Figure 3A), which might reflect an increase in transcriptional plasticity and control.

**Figure 4:**
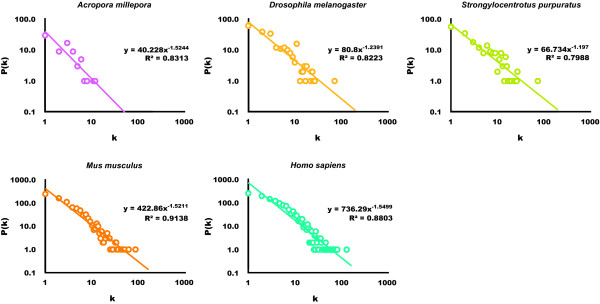
**Scale-free property of the transcriptional network**. Degree distributions of TF networks in all five species asymptotically follow a power law.

TF interaction network of *A. millepora *also showed scale-free property despite relatively small number of TFs compared to other species (Figure 4).

### Evidence for Taxonomically Restricted Interactions (TRIs)

In evolutionary biology the concept of Taxonomically Restricted Genes (TRGs) is associated with those genes that are restricted to particular species and are responsible for species-specific phenotypes and/or lineage-specific adaptations [41]. These genes usually evolve and adapt as a consequence of the specific environment and lifestyle of the organism and are more common in organisms that live in extreme environmental conditions. Although we do not debate the existence of such TRGs, we identified cases where the transcriptional network differs between specific organisms. Most importantly this does not correlate with the complexity of the organism but rather with its adaptation to a particular environment. Consequently, we propose that modulation of transcriptional networks might be a prime mechanism for species-specific adaptations. For example in Figure 3B, we report cases where specific interactions were gained or lost in specific species during evolution. In other words, we can identify what we like to call "Taxonomically Restricted Interactions" (TRIs). This new concept is also supported by the network expansion that we see in the mammalian lineage (Figure 3A). Overall, the concept might give a better explanation how an organism adapts to specific environments than merely considering a set of TRGs. Note that we anticipate plentiful examples of TRIs if whole protein interactomes were to be compared. Our results also support the notion that transcriptional networks evolve by gene duplication followed by gain or loss of specific interactions [42]. For example, the subnetworks in Figure 3B are typical examples of lineage-specific network expansions by gene duplication and interaction retention.

We also identified an interesting subnetwork that is composed of the oncogene MAX (Figure 3B network 7). In mammals, this network is composed of the homodimer of the oncogene MAX. In contrast, *A. millepora *retained a paralog forming a redundant system that is composed of two homodimers (Figure 3B). We argue that this paralog diverged from its ortholog sister as inferred from the alignment of the HLH domain and the evolutionary tree (Figure 3C and 3D).

## Conclusions

The here presented analysis scratches just the surface of our understanding of the complex structure of the transcriptional network in *A. millepora*. This study is a first attempt toward understanding the structure of the transcription factor networks in corals despite the paucity of - omics-level datasets including genome sequences. Contrary to the apparent simplicity of *A. millepora*, its gene repertoire and more importantly its transcriptional network are not so different from those of higher organisms and definitely not less complex than the ones of other model organisms such as *D. melanogaster*, making Scleractinian corals a candidate for a new model organism.

## Methods

### Coral protein sequence identification

To identify *A. millepora *TFs, ORFs were firstly searched in *A. millepora *transcriptome data [13] and then all possible coding sequences were translated using GetORF with default parameters [19]. Coding sequences from each contig/singleton were queried against the NCBI non-redundant (nr) protein database, and then best hits were chosen using BLASTP program. If there was no hit to nr database, the longest coding sequence (CDS) of the contig/singleton was chosen as a final CDS. Among 104,005 contigs from the transcriptome data, 19,840 and 49,320 sequences were chosen by above two approaches, respectively.

### Identification of TFs with DNA binding domains

To identify transcription factors of five analyzed species, we used 147 Pfam domains that bind DNA in a sequence-specific manner [16]. InterProScan [21] was used to search Pfam domains in protein sequences of five organisms, and then a protein was regarded as a TF if it contains at least one DNA binding domain. This resulted in 359, 1,047, 839, 1,462, 1,885 TFs for *A. millepora *(AM), *D. melanogaster *(DM), *S. purpuratus *(SP), *M. musculus *(MM), and *H. sapiens *(HS), respectively. To assess the accuracy of prediction, a 'known' human TF set [43] was downloaded. 1,443 known TFs and 1,405 predicted TFs were obtained after converting TF lists to Entrez gene IDs. Among the known TFs, 1,226 were predicted correctly and 217 did not have any sequence-specific DNA binding domains. 179 predicted TFs did not overlap with known TFs. Positive predictive value, ratio of true positives to sum of true and false positives, was 87.26%.

### Analysis of domain architecture

We searched the Pfam domain annotation for these 359 coral transcription factors, and recorded all types of domains they contained. The results showed that the total of all identified TFs consisted of 60 types of protein family domains.

To compare across species we applied an arcsine transformation to above data [25]. The arcsine transformation converts a binomial random variable into one that is nearly normal and whose variance depends very little on the probability parameter, by taking the arcsine of the square root of the abundance value for each functional family domain.

If *X *is a binomial random variable with parameters *n *and *p*, then  is the arcsine transformation of *Χ*.

We further applied a Fisher's statistical test to measure how significantly different the five organisms are, based on their protein family domain composition. The contingency table compiled data from the top ten Pfam domains. In addition, we conducted pairwise comparisons to cluster organisms based on their domain composition. We constructed a phylogenetic tree based on the top ten most abundant protein domain families (Figure 2).

### Ortholog identification

Identification of orthologous TFs is the essential part of our approach. We therefore employed the Inparanoid algorithm that was specifically designed for identifying true orthologs solely based on the protein sequence [29,30]. Protein sequences of analyzed species were downloaded from NCBI (SP, MM, and HS) and Ensembl (DM). Coral protein sequences were obtained as described above. Inparanoid algorithm was applied for each species pair and orthologous proteins were retrieved.

### Inference of interlogs

Based on the assumption that if two proteins interact in one organism, their orthologs in other organisms will also interact with each other [18], we inferred interacting orthologs across species, i.e. interlogs, using available TF interaction data. 5,238 human and 1,145 mouse TF interactions data were obtained from mammalian 2 hybrid assay [1]. 45 fruitfly TF interaction data was additionally downloaded from DIP [17]. For each known interacting TF pair (A-B), we sought all orthologs of A (A*_i1_*, A*_i2_*, ···, A*_ip_*, ···, and A*_iP_*, where 1≤*p*≤*P*) and B (B*_i1_*, B*_i2_*, ···, B*_iq_*, ···, and B*_iQ_*, where 1≤*q*≤*Q*) in organism *i *using Inparanoid result. All possible pairs of A*_ip _*and B*_iq _*were then regarded as interlogs. In the case that an interaction did not have at least one DNA binding domain, this interaction was not considered. Finally, 3,985 interlogs were inferred using this approach, and the total number of interactions including source and inferred interactions were 5,509; 2,323; 524; 599 and 134 for human, mouse, sea urchin, fruitfly, and coral, respectively (see Additional files 7, 8, 9, 10, and 11).

### Conserved TF interactions

Essential proteins and interactions are usually conserved across many species [27,28]. We therefore aligned protein networks of above five model organisms in order to find such elements, and sought conserved interactions across five organisms (see Additional files 12, 13, 14, 15, and 16).

## List of abbreviations

TF: transcription factor; ORF: open reading frame; TRG: Taxonomically Restricted Genes; TRI: Taxonomically Restricted Interactions; CDS: coding sequence; AM: *Acropora millepora*; DM: *Drosophila melanogaster*; SP: *Strongylocentrotus purpuratus*; MM: *Mus musculus*; HM: *Homo sapiens*.

## Authors' contributions

TR1 (Taewoo Ryu), CHM, CRV, and TR2 (Timothy Ravasi) designed the research; TR1 and TB prepared data used in the study; TR1 and CHM performed data analysis; TR1, CHM, CRV, and TR2 drafted the manuscript; All authors read and approved the final manuscript.

## Supplementary Material

Additional file 1 **Table S1**. List of TFs in coral defined by domains composition. Click here for file

Additional file 2 **Table S2**. List of TFs in fruitfly defined by domains composition. Click here for file

Additional file 3 **Table S3**. List of TFs in sea urchin defined by domains composition. Click here for file

Additional file 4 **Table S4**. List of TFs in mouse defined by domains composition. Click here for file

Additional file 5 **Table S5**. List of TFs in human defined by domains composition. Click here for file

Additional file 6 **Table S6**. The conserved domains in the species-specific TFs repertoires. Click here for file

Additional file 7 **Table S7**. TF-TF interactions in coral. Click here for file

Additional file 8 **Table S8**. TF-TF interactions in fruitfly. Click here for file

Additional file 9 **Table S9**. TF-TF interactions in sea urchin. Click here for file

Additional file 10 **Table S10**. TF-TF interactions in mouse. Click here for file

Additional file 11 **Table S11**. TF-TF interactions in human. Click here for file

Additional file 12 **Table S12**. Conserved coral TF-TF interactions across the five species. Click here for file

Additional file 13 **Table S13**. Conserved fruitfly TF-TF interactions across the five species. Click here for file

Additional file 14 **Table S14**. Conserved sea urchin TF-TF interactions across the five species. Click here for file

Additional file 15 **Table S15**. Conserved mouse TF-TF interactions across the five species. Click here for file

Additional file 16 **Table S16**. Conserved human TF-TF interactions across the five species. Click here for file
