# Supplementary material for: Unexpected complexity of the Reef-Building Coral Acropora millepora transcription factor network
Source: BMC Syst Biol. 2011 Apr 28;5:58. doi: 10.1186/1752-0509-5-58 (PMC3096595; doi:10.1186/1752-0509-5-58)
Supplement: Additional file 6 — Table S6. The conserved domains in the species-specific TFs repertoires. [file 1752-0509-5-58-S6.PDF]

TABLE S6: NUMBER OF PFAM PROTEIN DOMAINS ACROSS SPECIES

Pfam Protein Domains	Coral	Sea Urchin	Fruit Fly	Mouse	Human	Total
**PF00008**	0	2	1	0	0	3
**PF00010**	35	76	75	113	134	433
**PF00017**	0	2	0	10	10	22
**PF00023**	0	4	8	4	6	22
**PF00025**	0	1	0	0	0	1
**PF00046**	76	187	188	281	264	996
**PF00053**	0	2	0	0	0	2
**PF00059**	0	2	0	0	0	2
**PF00067**	0	2	0	0	0	2
**PF00071**	0	2	0	0	0	2
**PF00078**	0	1	0	0	0	1
**PF00090**	0	2	0	0	0	2
**PF00096**	110	248	414	614	900	2286
**PF00097**	0	0	5	8	12	25
**PF00098**	0	2	0	4	2	8
**PF00100**	0	0	1	0	0	1
**PF00104**	4	56	39	60	101	260
**PF00105**	8	56	44	60	102	270
**PF00147**	0	2	0	0	0	2
**PF00149**	0	1	0	0	0	1
**PF00157**	4	7	13	17	17	58
**PF00168**	0	1	0	0	0	1
**PF00170**	10	14	23	49	62	158
**PF00176**	0	0	0	1	1	2
**PF00178**	12	22	18	29	45	126
**PF00226**	0	2	0	0	2	4
**PF00228**	0	0	1	0	0	1
**PF00249**	0	5	4	7	7	23
**PF00250**	21	44	30	46	54	195
**PF00271**	0	0	0	1	1	2
**PF00292**	8	11	20	12	25	76
**PF00313**	6	9	6	9	12	42
**PF00319**	3	4	8	6	8	29
**PF00320**	3	10	12	14	20	59
**PF00400**	0	0	0	3	2	5
**PF00412**	2	11	13	17	15	58
**PF00431**	0	1	0	0	0	1
**PF00439**	1	9	6	7	11	34
**PF00447**	1	2	4	5	11	23
**PF00498**	1	2	6	2	2	13
**PF00505**	0	1	0	0	1	2
**PF00520**	0	2	0	0	0	2
**PF00526**	0	0	1	0	0	1
**PF00530**	0	1	0	0	0	1
**PF00531**	0	1	0	2	4	7
**PF00533**	0	0	0	1	4	5
**PF00536**	0	1	2	5	4	12
**PF00554**	1	5	11	15	24	56

TABLE S6: NUMBER OF PFAM PROTEIN DOMAINS ACROSS SPECIES

Pfam Protein Domains	Coral	Sea Urchin	Fruit Fly	Mouse	Human	Total
**PF00569**	0	3	1	3	4	11
**PF00605**	4	4	0	13	17	38
**PF00612**	0	0	4	2	2	8
**PF00622**	0	0	0	1	0	1
**PF00628**	1	8	12	12	20	53
**PF00641**	0	0	0	1	1	2
**PF00642**	0	2	0	0	0	2
**PF00643**	0	2	0	2	0	4
**PF00646**	0	0	0	1	1	2
**PF00651**	0	2	75	54	68	199
**PF00751**	5	4	6	6	9	30
**PF00773**	0	2	0	0	0	2
**PF00853**	0	3	6	8	6	23
**PF00855**	0	0	1	0	0	1
**PF00856**	0	0	0	4	7	11
**PF00870**	2	2	2	13	10	29
**PF00904**	0	0	28	0	1	29
**PF00907**	11	10	11	22	25	79
**PF00929**	0	2	0	1	1	4
**PF00989**	2	14	10	14	20	60
**PF01017**	0	2	5	10	10	27
**PF01048**	0	2	0	0	0	2
**PF01056**	2	1	0	5	5	13
**PF01082**	0	1	0	0	0	1
**PF01131**	0	0	1	1	1	3
**PF01167**	3	4	3	7	7	24
**PF01210**	0	0	1	0	0	1
**PF01285**	2	2	7	6	6	23
**PF01342**	2	2	2	8	12	26
**PF01352**	0	0	0	242	381	623
**PF01381**	1	2	2	1	2	8
**PF01388**	0	2	2	5	8	17
**PF01396**	0	0	1	1	1	3
**PF01409**	0	0	0	1	1	2
**PF01422**	2	12	4	3	5	26
**PF01424**	0	0	2	1	1	4
**PF01426**	0	4	3	4	6	17
**PF01428**	0	2	2	2	4	10
**PF01429**	0	2	4	2	2	10
**PF01448**	0	3	4	7	7	21
**PF01530**	0	5	2	8	5	20
**PF01586**	0	4	1	4	4	13
**PF01683**	0	0	1	0	0	1
**PF01751**	0	0	1	1	1	3
**PF01753**	0	0	2	1	1	4
**PF01754**	0	6	3	4	6	19
**PF01762**	0	1	0	0	0	1
**PF01833**	1	4	15	17	26	63

TABLE S6: NUMBER OF PFAM PROTEIN DOMAINS ACROSS SPECIES

Pfam Protein Domains	Coral	Sea Urchin	Fruit Fly	Mouse	Human	Total
PF01853	0	2	1	1	1	5
PF02023	0	0	0	43	63	106
PF02037	0	2	9	5	5	21
PF02045	1	1	1	2	2	7
PF02121	0	1	0	0	0	1
PF02135	2	2	1	2	3	10
PF02155	0	0	0	1	7	8
PF02159	0	0	0	1	4	5
PF02161	0	0	0	1	1	2
PF02165	0	0	0	1	3	4
PF02166	0	0	0	1	1	2
PF02172	0	2	1	2	3	8
PF02173	1	2	7	14	15	39
PF02178	0	12	28	12	17	69
PF02198	0	7	7	12	16	42
PF02204	0	2	1	1	1	5
PF02214	0	0	0	1	1	2
PF02257	4	8	3	8	9	32
PF02319	2	7	6	10	12	37
PF02338	0	2	0	1	1	4
PF02344	0	0	0	1	1	2
PF02363	0	0	1	0	0	1
PF02373	0	4	2	5	8	19
PF02375	0	2	2	5	8	17
PF02376	1	3	3	7	8	22
PF02757	0	0	1	0	0	1
PF02791	0	6	5	3	5	19
PF02818	0	0	0	0	1	1
PF02820	0	1	0	1	2	4
PF02852	0	2	0	0	0	2
PF02864	1	2	5	10	10	28
PF02865	0	2	3	10	10	25
PF02891	2	6	17	15	17	57
PF02892	0	5	7	1	4	17
PF02928	0	4	2	5	8	19
PF02944	0	4	26	0	0	30
PF03131	4	4	2	8	12	30
PF03154	0	2	0	1	3	6
PF03172	0	0	0	4	8	12
PF03184	0	4	0	1	1	6
PF03299	1	2	2	8	5	18
PF03343	2	8	2	1	1	14
PF03372	0	2	0	1	1	4
PF03403	0	2	0	0	0	2
PF03474	3	2	2	3	3	13
PF03529	0	0	0	2	3	5
PF03615	0	1	3	2	2	8
PF03712	0	1	0	0	0	1

TABLE S6: NUMBER OF PFAM PROTEIN DOMAINS ACROSS SPECIES

Pfam Protein Domains	Coral	Sea Urchin	Fruit Fly	Mouse	Human	Total
PF03792	1	2	3	5	5	16
PF03798	0	2	2	3	3	10
PF03826	4	25	9	16	16	70
PF03859	0	0	4	2	2	8
PF03867	0	0	1	0	0	1
PF03957	0	2	2	3	3	10
PF04032	1	0	0	0	0	1
PF04054	1	0	3	0	1	5
PF04055	0	2	0	0	0	2
PF04253	0	1	0	0	0	1
PF04516	2	2	8	7	9	28
PF04564	0	1	0	0	0	1
PF04589	0	0	0	4	4	8
PF04606	0	0	0	1	0	1
PF04617	0	0	0	4	4	8
PF04621	0	1	0	3	10	14
PF04704	0	0	0	6	4	10
PF04731	0	0	0	3	3	6
PF04734	0	2	0	0	0	2
PF04812	0	0	0	2	2	4
PF04813	0	0	0	1	1	2
PF04814	0	6	0	2	2	10
PF05001	0	0	0	1	1	2
PF05044	0	2	3	2	2	9
PF05110	0	2	7	5	5	19
PF05224	1	4	1	1	1	8
PF05225	0	10	25	2	1	38
PF05349	0	0	0	3	3	6
PF05485	0	0	4	0	0	4
PF05699	0	5	1	1	3	10
PF05729	0	3	0	0	0	3
PF05764	1	4	1	1	1	8
PF06001	0	2	1	2	3	8
PF06010	0	2	1	2	3	8
PF06220	0	0	0	0	2	2
PF06320	1	2	1	1	1	6
PF06529	0	0	0	1	1	2
PF06546	0	0	0	2	3	5
PF06573	1	0	0	1	0	2
PF06621	0	0	0	2	3	5
PF06809	0	0	1	0	0	1
PF06818	0	0	1	6	4	11
PF06831	0	0	0	1	1	2
PF06839	0	4	1	6	6	17
PF07093	1	2	1	1	3	8
PF07469	0	0	0	1	4	5
PF07479	0	0	1	0	0	1
PF07525	0	2	1	2	1	6

TABLE S6: NUMBER OF PFAM PROTEIN DOMAINS ACROSS SPECIES

Pfam Protein Domains	Coral	Sea Urchin	Fruit Fly	Mouse	Human	Total
**PF07527**	4	9	13	11	13	50
**PF07528**	0	2	0	0	0	2
**PF07645**	0	0	1	0	0	1
**PF07647**	0	2	0	7	4	13
**PF07690**	0	4	0	0	0	4
**PF07710**	0	0	0	13	6	19
**PF07716**	7	13	50	62	69	201
**PF07776**	0	0	96	1	2	99
**PF07992**	0	2	0	0	0	2
**PF08172**	0	2	0	0	0	2
**PF08265**	0	2	1	1	1	5
**PF08279**	0	0	0	1	1	2
**PF08383**	0	0	0	5	5	10
**PF08420**	1	1	2	3	5	12
**PF08429**	0	0	2	4	7	13
**PF08430**	0	2	2	3	4	11
**PF08447**	0	10	15	14	20	59
**PF08474**	0	0	0	6	2	8
**PF08504**	0	0	0	8	5	13
**PF08523**	1	2	2	1	2	8
**PF08563**	0	2	0	2	7	11
**PF08778**	0	0	0	1	1	2
**PF08781**	1	4	2	2	3	12
**PF08815**	0	0	0	1	4	5
**PF08828**	0	0	3	0	0	3
**PF08832**	0	0	0	1	4	5
**PF09030**	0	2	0	2	3	7
**PF09091**	0	0	0	1	1	2
**PF09329**	0	2	0	0	0	2
**PF09332**	0	2	0	0	0	2
**PF09354**	0	2	0	0	0	2
**PF09770**	0	2	0	0	0	2
**PF09804**	0	2	0	0	0	2
**PF10401**	0	2	0	0	0	2
**PF10408**	0	1	0	0	0	1
**PF10453**	1	2	0	0	0	3
**PF10513**	1	0	0	0	0	1
**PF10525**	0	2	0	0	0	2
**PF10537**	0	2	0	0	0	2
**PF10545**	0	4	0	0	0	4
	398	1223	1599	2369	3133	**8722**
